# Can 2009 herald a new era in preventing cervical cancers?

**DOI:** 10.4103/1477-3163.45315

**Published:** 2009-01-30

**Authors:** Bernice Robinson-Bennett

**Affiliations:** Department of Obstetrics and Gynecology, Division of Gynecologic Oncology, The Reading Hospital and Medical Center, Regional Cancer Center, Sixth Avenue and Spruce Street, West Reading, PA 19611, USA

Cervical cancer still remains the second leading cause of cancer related mortalities worldwide, despite significant strides made in its prevention and treatment over the last 50 years. In the United States alone, it is estimated that 3870 women died of cervical cancer in 2008 and it is anticipated that there will be 11,000 new cases in the new year. While the link between persistent infection with oncogenic Human Papillomavirus (HPV) and the incidence of cervical cancer is long known, the molecular processes involved in the multistep carcinogenic transformation is yet to be elucidated.

It is currently believed that there are approximately 15 high risk oncogenic HPV viral types. The HPV E6 and E7 proteins target the p53 and the retinoblastoma tumor suppressor genes, respectively. The major processes involved in cervical carcinogenesis include infection of the metaplastic cervical epithelium, persistence of the virus, progression to a precancerous lesion and, finally, invasion of the basement membrane that results in invasive cervical cancer. As many as 5-10 years can elapse from the time of persistent infection to the stage of clinically evident disease. It may be another decade from a precancerous lesion to the development of invasive cancer.

A recent advancement in the treatment of early cervical cancer is the fertility conserving procedure of radical trachelectomy. This involves removal of the cervix with the involved tumor and margins, while leaving the body of the uterus for carrying a pregnancy. This procedure was first described by Dargent in 1994 and it is gaining world wide acceptance. Successful pregnancy rates after radical trachelectomy are as high as 40-70%.[1] Prior to this, young women with cervical cancers were relegated to hysterectomy or radiation therapy that would render them infertile.

A major impediment in preventing cervical cancers is the lack of sensitive and specific techniques for early detection. The Papanicolaou smear for cervical cancer screening was introduced in 1949, prior to the known link between HPV and cervical cancer.[2] Since its advent, the incidence and subsequent mortality from cervical cancer has decreased by as much as 50-60%.[3] Even with this progress, there are still significant limitations associated with the Pap smear. Inadequate samples are reported in as many as 8% of the cases and the false negative rate can range from 15-30%.[4–6] There are multiple factors that can contribute to these rates including blood, bacteria or yeast contamination, as well as human error. In order to impose some regulatory control on the false negative rate, the Clinical Laboratory Improvement Act has mandated a re-screening of 10% of pap smears considered adequate but which were read as negative.

In an effort to improve the quality of the pap testing, a majority of the institutions have converted from conventional glass slide pap to liquid based pap, in which the cells are suspended in a medium to remove blood and debris, with the goal of decreasing the number of false negative or false positive or numerous low grade abnormalities of the cytological interpretation. Since then, we have also identified methodologies of identifying high risk oncogenic HPV viral types from low risk types. This information is now used in conjunction with the Pap smear analysis, to stratify patients needing further evaluation and or treatment.

Recent years have seen a burst of efforts to produce prophylactic vaccines for the prevention of cervical cancer. Prophylactic vaccines induce virus neutralizing antibodies, to protect against new infections; whereas, therapeutic vaccines are aimed at inducing a cellular immunity to already infected epithelial cells. There have been numerous phase II and III trials reporting close to 100% efficacy of HPV vaccines in HPV naïve individuals, in preventing precancerous cervical lesions and consequential cervical cancer. In June 2006, the first vaccine *Gardasil* was approved by the Food and Drug Administration (FDA) for the prevention of precancerous lesions. This is a quadrivalent vaccine, with protection against HPV 16, and 18, the two viral types that account for up to 70% of cervical cancer[7] and HPV 6, and 11, the two most prominent types causing genital warts. Other prophylactic HPV vaccines are awaiting FDA approval. Researchers are also looking into the prospects of therapeutic vaccination against cervical cancer.

Does the advent of these vaccinations mark the beginning of the end of the cervical cancer epidemic? Hopefully it does, but we have a long way to go.

The efficacy of the vaccine against moderate and severe precancerous lesion in all users was only 17%,[8] which highlights the importance of the timing of the vaccination, before sexual debut, and the need for continued screening programs. Additionally, there is still a plethora of unanswered questions around HPV vaccination, including the duration of effect and the need for any booster shot, implication of vaccinating males, vaccine cross protection against nonvaccine types and the cost of vaccination. The long latency period of the virus cannot be underestimated when considering the prospects at eradicating cervical cancer. It will take the next several decades to evaluate the true impact of the vaccination against cervical cancer. This can only be accomplished through longitudinal population trials such as the Nordic cohort, which anticipates results on the incident rates of cervical cancer and severe precancerous lesions in 2020.[9]

One single major difficulty in identifying high risk groups and early lesions is the significant barrier to screening. It is well known that the population most susceptible to acquisition of the disease is the same group that will not be screened or that will not obtain the vaccine. The other paramount issue is the lack of effective treatments in recurrent cervical cancers, besides those patients who are deemed resectable or those who are naïve to radiation. The lack of these treatments probably reflects the lack of a deeper understanding to molecular aspects in the carcinogenesis model. Future studies with proteomics and genomics may shed light on these processes and may mine new therapeutics in the management to of cervix cancer.

The most important and yet difficult aspect of prevention of cervical cancer is the understanding of its genesis - both at the etiological and molecular level. These are still exciting times for ongoing research in cervical cancer.

January is the international cervical cancer awareness month. Cervical cancer is one of the preventable cancers and is a serious public health issue. The Journal of Carcinogenesis is committed to promoting the efforts of the international community of researchers and public health officials in bringing cancer awareness to the public. The journal welcomes manuscripts that shed light on cervical carcinogenesis and its prevention.
